# In vitro effects of tropisetron and granisetron against *Echinococcus granulosus *(*s.s.*) protoscoleces by involvement of calcineurin and calmodulin

**DOI:** 10.1186/s13071-021-04691-9

**Published:** 2021-04-12

**Authors:** Mohammad Reza Shiee, Eshrat Beigom Kia, Farzaneh Zahabiun, Mahmood Naderi, Elahe Motevaseli, Shahram Nekoeian, Majid Fasihi Harandi, Ahmad Reza Dehpour

**Affiliations:** 1 Department of Medical Parasitology and Mycology, School of Public Health, Tehran University of Medical Sciences, Tehran, Iran; 2 Cell-Based Therapies Research Center, Digestive Disease Research Institute, Tehran University of Medical Sciences, Tehran, Iran; 3 Department of Molecular Medicine, School of Advanced Technologies in Medicine, Tehran University of Medical Sciences, Tehran, Iran; 4 Research Center for Hydatid Disease in Iran, Kerman University of Medical Sciences, Kerman, Iran; 5 Experimental Medicine Research Center, Tehran University of Medical Sciences, Tehran, Iran; 6 Department of Pharmacology, School of Medicine, Tehran University of Medical Sciences, Tehran, Iran

**Keywords:** Tropisetron, Granisetron, Cyclosporine A, Albendazole sulfoxide, *Echinococcus granulosus*, Calcineurin, Calmodulin, In vitro

## Abstract

**Background:**

Cystic echinococcosis (CE) is a disease caused by the larval stage of *Echinococcus granulosus* sensu lato  (*s.l.*). The treatment of CE mainly relies on the use of benzimidazoles, which can commonly cause adverse side effects. Therefore, more efficient treatment options are needed. Drug repurposing is a useful approach for advancing drug development. We have evaluated the in vitro protoscolicidal effects of tropisetron and granisetron in* E. granulosus* sensu stricto (*s.s.*) and assessed the expression of the calcineurin (CaN) and calmodulin (CaM) genes, both of which have been linked to cellular signaling activities and thus are potentially promising targets for the development of drugs.

**Methods:**

Protoscoleces (PSC) of *E. granulosus *(*s.s.*) (genotype G1) obtained from sheep hepatic hydatid cysts were exposed to tropisetron and granisetron at concentrations of 50, 150 and 250 µM for various periods of time up to 10 days. Cyclosporine A (CsA) and albendazole sulfoxide were used for comparison. Changes in the morphology of PSC were investigated by light microscopy and scanning electron microscopy. Gene expression was assessed using real-time PCR at the mRNA level for *E. granulosus *calcineurin subunit A (Eg-CaN-A), calcineurin subunit B (Eg-CaN-B) and calmodulin (Eg-CaM) after a 24-h exposure at 50 and 250 µM, respectively.

**Results:**

At 150 and 250 µM, tropisetron had the highest protoscolicidal effect, whereas CsA was most effective at 50 µM. Granisetron, however, was less effective than tropisetron at all three concentrations. Examination of morphological alterations revealed that the rate at which PSC were killed increased with increasing rate of PSC evagination, as observed in PSC exposed to tropisetron. Gene expression analysis revealed that tropisetron at 50 μM significantly upregulated Eg-CaN-B and Eg-CaM expression while at 250 μM it significantly downregulated both Eg-CaN-B and Eg-CaM expressions; in comparison, granisetron decreased the expression of all three genes at both concentrations.

**Conclusions:**

Tropisetron exhibited a higher efficacy than granisetron against *E. granulosus *(*s.s.*) PSC, which is probably due to the different mechanisms of action of the two drugs. The concentration-dependent effect of tropisetron on calcineurin gene expression might reflect its dual functions, which should stimulate future research into its mechanism of action and evaluation of its potential therapeutical effect in the treatment of CE.

**Graphic Abstract:**

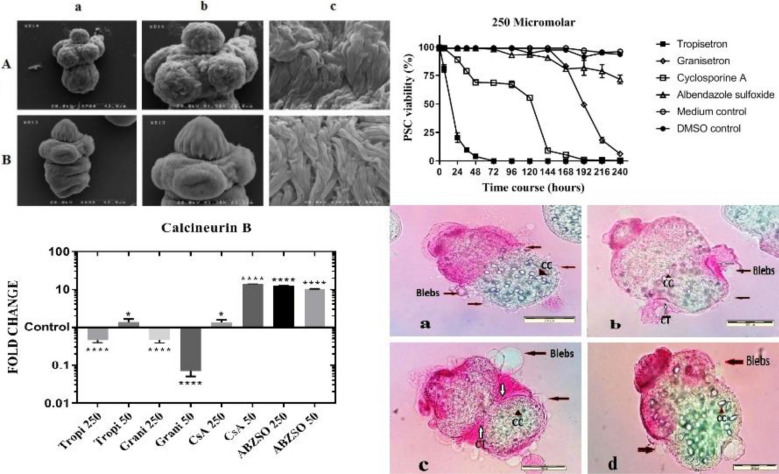

## Background

Human cystic echinococcosis (CE) is a global zoonosis caused by the larval or metacestode stage of the dog tapeworm *Echinococcus granulosus* sensu lato (*s.l.*) [1]. Treatment of CE is often expensive and complicated, requiring extensive surgery and/or prolonged drug therapy [2]. There are four options for the treatment of CE: (i) percutaneous treatment of the hydatid cysts with the PAIR (puncture, aspiration, injection, re-aspiration) technique; (ii) surgery; (iii) anti-parasitic drug treatment; and (iv) the “watch and wait” approach [2]. The benzimidazole (BMZ) compounds albendazole and mebendazole have been the cornerstone of drug-based CE treatment [1]. These are typically administered to inoperable patients with liver or lung CE and patients with multiple cysts in two or more organs, or peritoneal cysts [3]. Benzimidazoles are also used to prevent recurrence following surgery or PAIR [4]. However, long-term use of these compounds is linked to the development of adverse effects, including hepatotoxicity, severe leucopenia, thrombocytopenia and alopecia. Therefore, new and more efficient treatment options are urgently needed [5].

Drug repurposing is a useful strategy to accelerate drug development due to the associated lower costs, reduced risk of treatment failure and feasible marketing based on the availability of preclinical data. This strategy enables not only pharmaceutical companies but also public sector researchers to engage in drug discovery and development efforts [6]. Tropisetron and granisetron are selective receptor antagonists of serotonin 3 (5-hydroxytryptamine 3 [5-HT_3_]) that are widely used as effective, well-tolerated and safe agents to counteract chemotherapy-induced emesis [7]. However, tropisetron, but not granisetron, has been shown also to inhibit the phosphatase activity of calcineurin (CaN), an enzyme that is pivotal in activating transcriptional factors responsible for immune/inflammatory axis regulation at both the transcriptional and protein levels and to target CaN in a receptor-independent fashion [8]. Moreover, it was found that tropisetron specifically inhibits both interleukin 2 (IL-2) gene transcription and IL-2 synthesis in stimulated T cells and inhibits early and late events in T-cell activation [9]. Nevertheless, it remains undetermined whether tropisetron directly inhibits CaN or whether it acts* via* CaN-interacting molecules such as immunophilins [8]. The identification of tropisetron mechanisms other than the antagonism of 5-HT_3_ receptors could form a basis for new drug development [8].

Calcineurin, known as protein phosphatase 2B (PP2B), is a Ca^2+^-calmodulin (CaM)-activated serine/threonine protein phosphatase involved in different signaling pathways. It is a heterodimeric protein consisting of the catalytic subunit CaN A (CaN-A) and the Ca^2+^-binding subunit, CaN B (CaN-B) [10]. Calcineurin plays a key role in the coupling of local or global calcium signals, which in turn control immediate cellular responses and modify gene transcription [11]. The biological functions of CaN have been studied in several organisms, including parasitic helminths such as *Schistosoma mansoni*, *Hymenolepis microstoma* and *H. diminuta*, in which CaN is involved in ion homeostasis and regulation of exocytosis [12, 13]. CaN has also been characterized in larval stages of *E. granulosus *(*s.l.*), and CaN-A has been immunolocalized in the cytoplasm of tegumental cells, suckers and excretory bladder of protoscoleces (PSC) [14].

The CaM gene is also expressed in the tegument tissue and parenchymal region of PSC [15, 16]. In terms of evolution, CaM is one of the most ancient proteins in eukaryotes. The functions of CaM include Ca^2+^ binding and conversion of Ca^2+^ signals through downstream proteins to regulate various physiological processes, such as muscle contraction, metabolism and cell motility [17]. Calcineurin and CaM have been linked to the regulation of the life cycle, development and cellular signaling activities, thus being potentially promising targets for the development of drugs that block the invasion of parasites and limit their growth [14, 15].

With the aim to evaluate new compounds against *E. granulosus* sensu stricto (*s.s.*) PSC by means of drug repurposing, this study was designed to evaluate the effectiveness of two safe and clinically available drugs: tropisetron and granisetron. Tropisetron, which has a dual molecular signaling pathway [9], and granisetron, both of which are selective 5-HT_3_ receptor antagonists [18], were used in vitro in comparison with the immunosuppressant drug cyclosporine A (CsA), which is a non-competitive inhibitor of CaN phosphatase activity [19]. CsA binds to cyclophilin proteins and prevents conformational changes in the CaN-A subunit, which is an enzyme involved in T-cell activation [20]. The effects of CsA on *E. granulosus *(*s.l.*) PSC have already been reported [21], but the exact mechanism of action remains unknown. Albendazole sulfoxide (ABZSO), the protoscolicidal metabolite of albendazole [1], was also included in the evaluation as an already known effective compound currently used in CE treatment [22]. Moreover, changes in *E. granulosus *CaN (Eg-CaN) and *E. granulosus *CaM (Eg-CaM) gene expression at the mRNA level were analyzed and the morphological changes in the PSC were evaluated using light and scanning electron microscopy (SEM).

## Methods

### Collection of PSC

Sheep unilocular hepatic hydatid cysts were provided by abattoirs from the Tehran Province, Iran, and immediately transferred to the Helminthology Laboratory at the School of Public Health, Tehran University of Medical Sciences. Under aseptic conditions, each hydatid cyst was cut open, and the solution containing PSC was aspirated by a syringe and then allowed to settle in a 50-ml Falcon tube. After precipitation of the parasites, the supernatant was discarded and the PSC were washed three times with sterile phosphate-buffered saline (PBS) (pH 7.2). The viability of the PSC was assessed using an exclusion test [23] that consisted of observing PSC impermeability to 0.1% eosin solution using light microscopy. Red-stained PSC were considered to be dead, whereas colorless PSC were considered to be viable. The drug assessment tests involved numerous PSC with > 90% viability and belonging to *E. granulosus *(*s.s.*) G1 genotype. Genotype identification was performed on the samples of PSC retrieved from every appropriate pellet (with > 90% viable PSC) and according to the procedure described below.

### Genotype determination of PSC

Total genomic DNA of PSC was extracted using the High-Pure PCR Template Preparation Kit (Roche, Mannheim, Germany) according to the manufacturer’s instructions. The cytochrome* c *oxidase subunit 1 (*cox*1) gene of the mitochondrial genome was amplified and sequenced to reveal the genotype of PSC isolated from the hydatid cysts. The PCR was carried out as described by Bowles et al. [24] using the primers JB3 (5′-TTT TTT GGG CAT CCT GAG GTT TAT-3′) and JB4.5 (5′-TAA AGA AAG AAC ATA ATG AAA ATG-3′), and the PCR product was sequenced using primers JB3 and JB4.5 at Bioneer Corporation (Daejeon, South Korea). The obtained sequences (amplicon size: 366 bp) were assembled using Chromas v.2.6.4 (Technelysium Pty Ltd., Brisbane, Queensland, Australia) and compared with sequences previously deposited at the National Center for Biotechnology Information (NCBI) using the BLAST tool (http://www.ncbi.nlm.nih.gov/blast).

### Culture of *E. granulosus *(*s.s.*) PSC

In vitro culture of the collected and washed PSC was as previously described [25]. Briefly, the PSC were transferred from the Falcon tube into filter-capped 75-cm^2^ cell culture flasks containing culture medium [Dulbecco's Modified Eagle Medium (DMEM) containing glutamax, d-glucose (4.5 g/l), HEPES (15 mM), sodium pyruvate, NaHCO_3_ (Gibco, New York, NY, USA) and 1% penicillin–streptomycin (Biosera, Nuaille, France), with 0.5 µg amphotericin B solution (Caisson Laboratories Inc., North Logan, UT, USA)], supplemented with 10% fetal calf serum (Biosera, Nuaille, France). Prior to initiation of the in vitro drug assay, the culture medium containing the PSC was maintained in an incubator at 37 °C and 5% CO_2_ for 24 h to ensure that the medium was not contaminated with fungal or bacterial infections.

### In vitro drug assays

With regard to the drug mixture preparations, tropisetron monohydrochloride (CAS no.: 105,826–92-4, Product no.: T104; Sigma-Aldrich, St. Louis, MO, USA) and granisetron hydrochloride (CAS no.: 107,007–99-8, Product no.: G3796; Sigma-Aldrich China, Shanghai, China), which are soluble in water, were diluted directly in the culture medium. However, CsA (CAS no.: 59865-13-3, Product no.: 30024, Sigma-Aldrich USA) and ricobendazole (ABZSO) (CAS no: 54029-12-8, Product no: 19953; Sigma-Aldrich China) were first dissolved in dimethyl sulfoxide (DMSO)(CAS no.: D8779, Product no: 67-68-5, Sigma-Aldrich USA) at an amount of < 0.3%, which has no deleterious effect on the parasite [26], and then diluted in the culture medium. Treatment of the PSC was initiated 1 day after initiation of in vitro culture. For all drugs, three final concentrations were prepared:50, 150 and 250 µM. Additionally, two cell culture flasks containing non-treated PSC were used as control cultures; one with culture medium only, and the other supplemented with DMSO. All treated PSC and the two controls were dispersed in the flasks at approximately equal numbers (*n* = 25,000), with medium change on day 5 of the initiation of the drug assessment. Protoscolex viability assessment following initiation of drug exposure was performed at 1-h inspections during the first 12 h and then at 24-h intervals up to the tenth day. The viability of the PSC was evaluated after gentle mixing of the solutions using an eosin 0.1% exclusion test [23] with microscopic observation of change in the color of the PSC. Viability was calculated as the proportion of viable PSC among the total number of PSC in ten microscopic fields at ×10 magnification. Each run of drugs tests was carried out in three replicates per treatment condition and repeated three times. After 24 h of drug exposure, some samples of treated PSC were harvested from the preparations containing 250 µM of the respective drug for SEM, and from those containing 50 and 250 μM for RNA extraction and comparison with the two controls.

### Scanning electron microscopy

Some specimens of PSC treated with tropisetron, granisetron, CsA and ABZSO at 250 µM for 24 h, as well as controls (medium control and DMSO control) were fixed with 2.5% glutaraldehyde in sodium cacodylate buffer for 24 h at 4 °C. After several rounds of washing in cacodylate buffer, samples were first fixed in 2% OsO4 in cacodylate buffer, then extensively washed in distilled water, dehydrated by sequential incubations in increasing concentrations of ethanol (50–100%), immersed in hexamethyldisilazane and air-dried under a fume hood [27]. The samples were then sputter-coated with gold (thickness: 100 Å) and observed with a scanning electron microscope (field emission-SEM; Hitachi model S-4160; Hitachi Ltd., Tokyo, Japan) operating at 20 kV. Finally, for each sample, four regions were visualized: (i) complete protoscolex (×500 magnification); (ii) sucker and rostellum (×1100 magnification); (iii) microtriches (×30,000 magnification); and (iv) soma region (×30,000 magnification).

### Molecular analysis

#### Gene identification and primer design

Calcineurin genes of subunit A (Eg-CaN-A) and B (Eg-CaN-B) and Eg-CaM were selected as the main genes for assessment, and glyceraldehyde 3-phosphate dehydrogenase (GAPDH) was chosen as reference gene. The sequences of these genes were obtained from two references [28, 29] available in the NCBI (accession no.: Xm_024493804, Xm_024491779, Xm_024490475 and Xm_024494574) or GENDB (accession no.: EgrG_000601200, EgrG_000454300, EgrG_000491400 and EgrG_0002546000) databases. Primers (Table 1) were designed using Allele ID (Premier Biosoft, Palo Alto, CA, USA) and MEGA v.7 and Gene Runner v.5.0.59 software and synthesized by Metabion International AG (Planegg, Germany)**.**

**Table 1:** Genes and corresponding primers for the gene expression analysis of *Echinococcus granulosus * (*s.s.*) protoscoleces exposed to the various drugs

Gene	Forward primer (5′–3′)	Reverse primer (5′–3′)	Amplicon length (bp)	PCR efficiency
Calcineurin A	GGACGACATTAGGCGGATTG	GCTGAGTAACTGAAGAAGTAGGAG	165	1.95
Calcineurin B	ATACGGATGGGAATGGCGA	CATCTTTAGAACCTGGAAAAGCTC	167	1.93
Calmodulin	ATCACCACCAAGGAATTAGGG	CGTCCCATTACCGTCTGC	108	1.91
GAPDH	TTCCACCACCTGCTCCTC	CCAAACTCATTATCGTACCAAGC	96	1.90–1.93

*GAPDH * Glyceraldehyde 3-phosphate dehydrogenase

#### Total RNA extraction and cDNA synthesis

Some PSC exposed to different drugs at 50 and 250 µM for 24 h, as well as controls (medium control and DMSO control) were placed in liquid nitrogen overnight and then physically shattered using syringes and needles and examined under an optical microscope to ensure that their walls were crushed. RNA was then extracted using the RNeasy Mini Kit (Qiagen, Hilden, Germany), according to the manufacturer’s instructions. RNA concentration was measured with a NanoDrop spectrophotometer (Thermo Fisher Scientific, Waltham, MA, USA). All concentrations were < 100 ng/ml, and absorption ratios at 280/260 nm were between 1.7 and 2.2. cDNA was then synthesized using the Quanti Nova Reverse Transcription Kit (Qiagen), according to the manufacturer’s instructions, and stored at − 20 °C until processed in real-time PCR assays.

#### Real-time PCR analyses

For the real-time PCR analyses, 1 μl of diluted (1:2) cDNA was used in a final reaction volume of 20 μl containing 10 μl of SYBR Premix Ex Taq II (2×), 0.4 ROX Reference Dye (Takara, Tokyo, Japan), 400 nM of each of the forward and the reverse primers, using the Step One Plus Real-Time PCR System (Applied Biosystems, Foster City, CA, USA). The reaction conditions included an initial activation step at 95 °C/2 min; denaturation at 95 °C/15 s, annealing at 60 °C/1 min, extension at 72 °C/30 s for 45 cycles. The point of quantification was adjusted according to the melting curves of the primers, and LinRegPCR v.2017.1 software (Ruijter et al., Amsterdam, the Netherlands; https://www.gene-quantification.de/LinRegPCR_help_manual_v11.0.pdf) was used to obtain data on PCR amplification efficiency.

### Statistical analysis

Data on changes in gene expression were analyzed using the REST 2009 software. Data on protoscolex viability and gene expression levels were presented as means ± standard deviations (SD) and illustrated using GraphPad Prism software (version 8.3.0; GraphPad Software, San Diego, CA, USA). Statistical analyses between groups used one-way analysis of variance (ANOVA) for gene expression analysis, and two-way ANOVA for viability evaluation with Dunnett post hoc tests. A *P* value of ≤ 0.05 was considered to be statistically significant.

## Results

### Sequence analysis

Protoscoleces processed for the investigations described in the following sections showed 100% identity with the *E. granulosus * (*s.s.*) G1 genotype (NCBI GenBank sequence HM563011) [30].

### Effect of drug exposure on *E. granulosus* (*s.s.*) PSC

Based on the results of the viability tests in both of the controls, the viability rate of PSC was > 95% after 240 h (10 days) of cultivation. Data on drug efficiency at each concentration are provided below.

#### Concentration of 250 μM

**Fig. 1:**
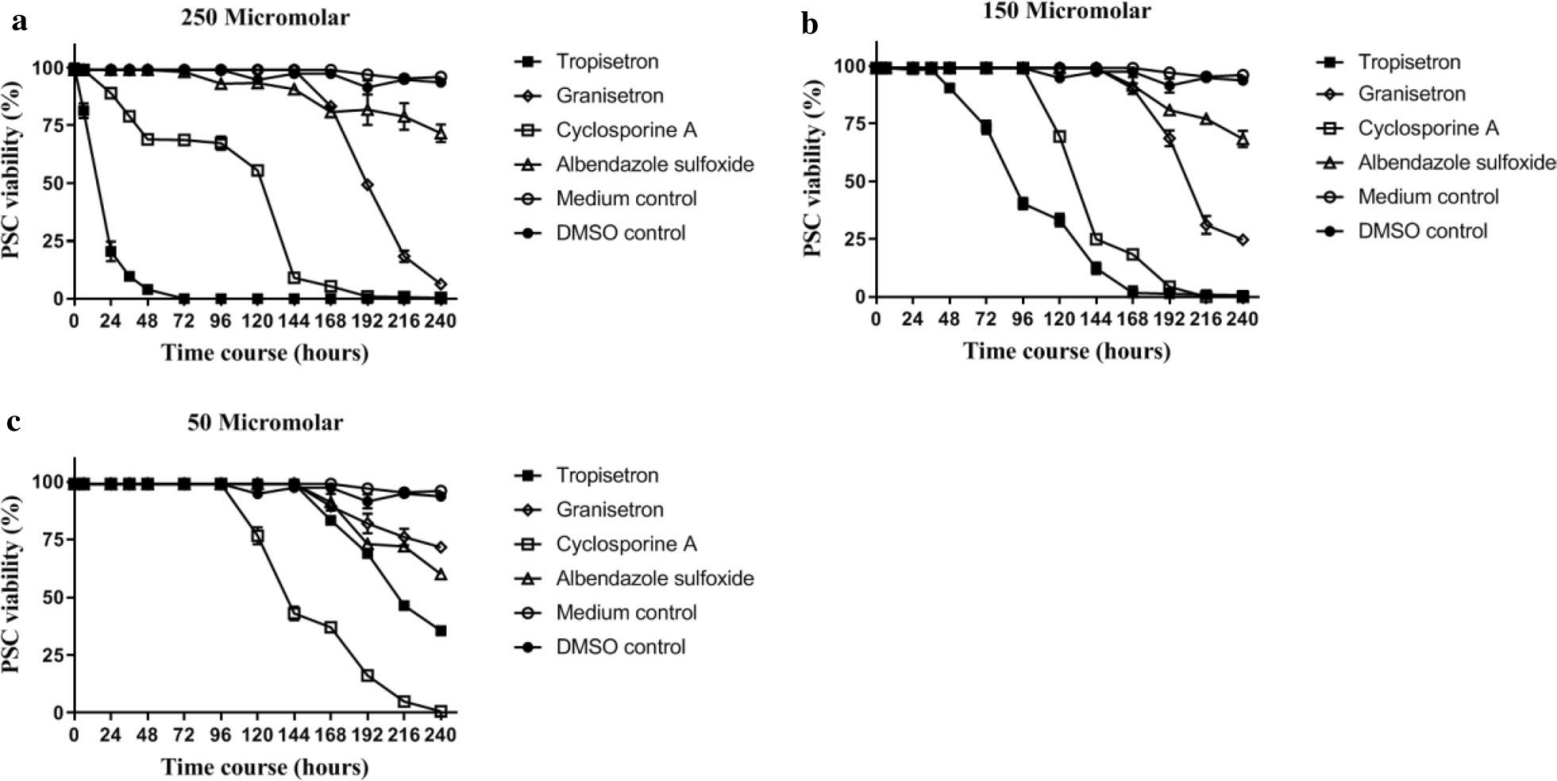
Trends in viability of *Echinococcus granulosus * (*s.s.*) protoscoleces (PSC) exposed to tropisetron, granisetron, cyclosporine A (*CsA*) and albendazole sulfoxide (*ABZSO*), for various exposure time courses ranging from 24 h to 240 h (10 days), at three concentrations: 250 μM (**a**), 150 μM (**b**) and 50 μM (**c**).* DMSO* Dimethyl sulfoxide

**Fig. 2:**
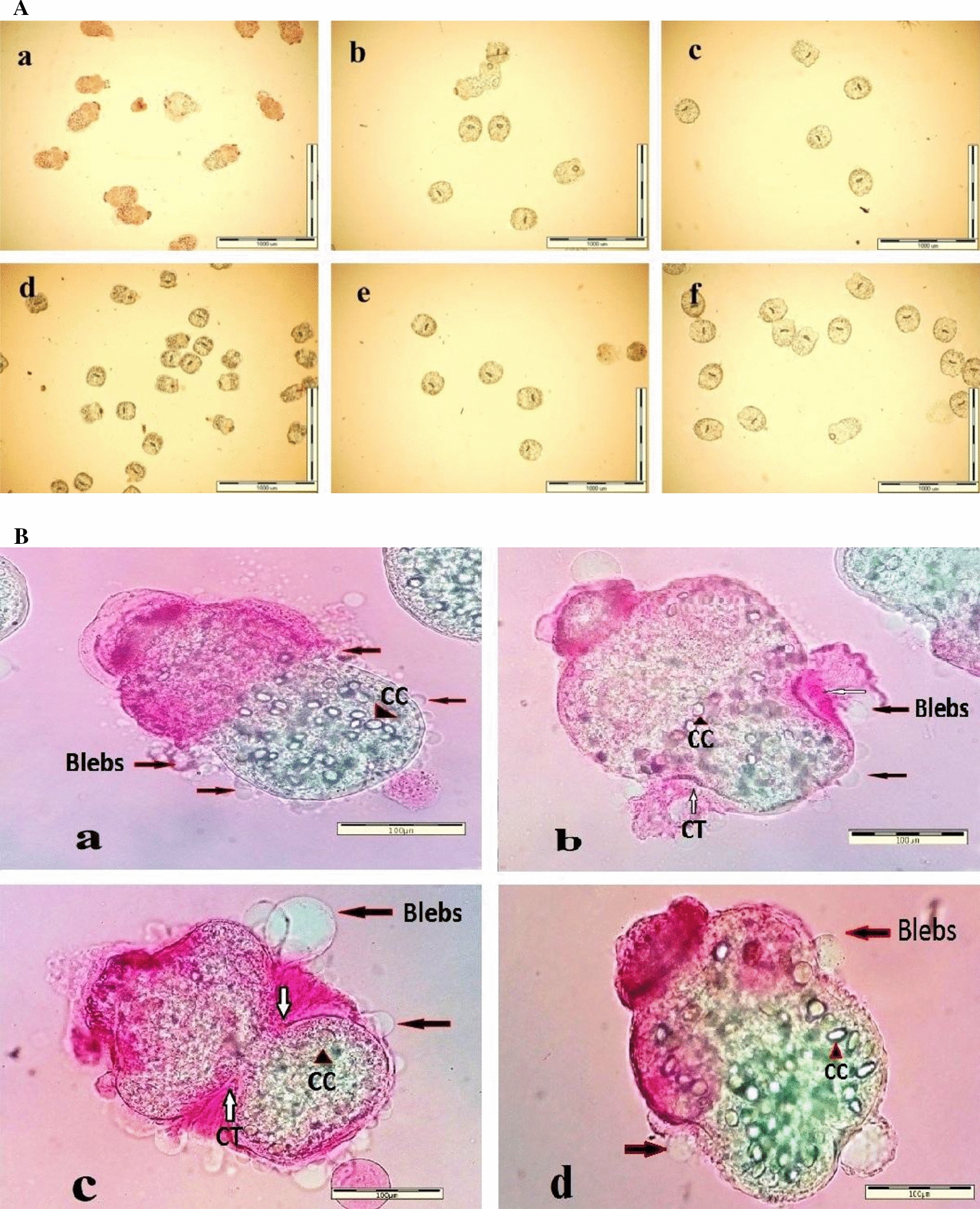
Optical microscope photographs of *E. granulosus * (*s.s.*) protoscoleces (*PSC*) exposed to different drugs and the non-exposed controls. **A** PSC after a 24-h exposure to 250 μM of tropisetron (**a**), granisetron (**b**), medium control (**c**), CsA (**d**), ABZSO (**e**), DMSO control (**f**); (magnification ×40). **B** PSC exposed to 250 μM of tropisetron after 24 h (**a**), granisetron after 175 h (**b**), CsA after 120 h (**c**), ABZSO after 140 h (**d**); (magnification ×400) . The presence of blebs in the tegument of the PSC (**a**,** b**,** c**,** d**), contraction of the tegument (*CT*) at the soma region (**b**,** c**), and calcareous corpuscles (*CC*) in parenchyma of the PSC (**a**,** b**,** c**, **d**) are visualized

The highest protoscolicidal effect at this concentration was observed for tropisetron. After 24 h of exposure to 250 μM tropisetron, 80% of the PSC were deemed non-viable (Fig. 1a). Protoscolex degeneration initiated from the scolex region and then spread to the soma region. Most of the PSC were evaginated (> 90% after 5 h) (Fig. 2Aa). Numerous blebs were observed on the tegument of the PSC, and shedding of the hooks was also noted (Fig. 2Ba).

More than 90% of the PSC exposed to granisetron exhibited evagination after 72 h. The protoscolicidal effect of granisetron was 92% after 168 h of exposure (Fig. 1a). Blebs were noted in the soma and in the scolex region (Fig. 2Bb). In addition, a large amount of the PSC had developed into the vesicular, evaginated form after 96 h (Fig. 2Bb).

With regard to CsA, a protoscolicidal effect was observed after 24 h of exposure. PSC consisted of both invaginated and evaginated forms (Fig. 2Ad), with > 90% of PSC having evaginated after 48 h. The tegument of the PSC contracted at the anterior soma region after 120 h of exposure, and blebs were observed to be bulging all over the tegument (Fig. 2Bc). More than 90% of the parasites were dead after 144 h.

Most of the ABZSO-treated PSC (> 90%) remained invaginated after 24 h (Fig. 2Ae). ABZSO-mediated alterations in the PSC were microscopically visible after 96 h, and after 240 h, the level of evagination had reached approximately 30% (Fig. 1a). Protoscoleces were permeable to eosin at the site of the scolex and soma region, and the presence of knob-like projections was observed at the soma region (Fig. 2Bd).

#### Concentrations of 150 and 50 μM

At concentrations of 150 and 50 μM, the time taken to trigger 90% evagination of the PSC was 48 and 96 h for tropisetron, 96 and 144 h for granisetron and 72 and 72 h for CsA, respectively. The evagination of PSC exposed to ABZSO was < 40% after 240 h.

Tropisetron began to exhibit protoscolicidal effects at 150 μM after 48 h, leading to death of 100% of PSC after 168 h. Exposure to CsA led to 100% mortality after 192 h, while exposure to granisetron and ABZSO led to 75 and 30% mortality, respectively, after 240 h (Fig. 1b). After incubation for 240 h with 50 μM of the studied drug, different mortality rates were observed:100% for CsA, 65% for tropisetron, 40% for ABZSO and 30% for granisetron (Fig. 1c).

### Scanning electron microscopy

**Fig. 3:**
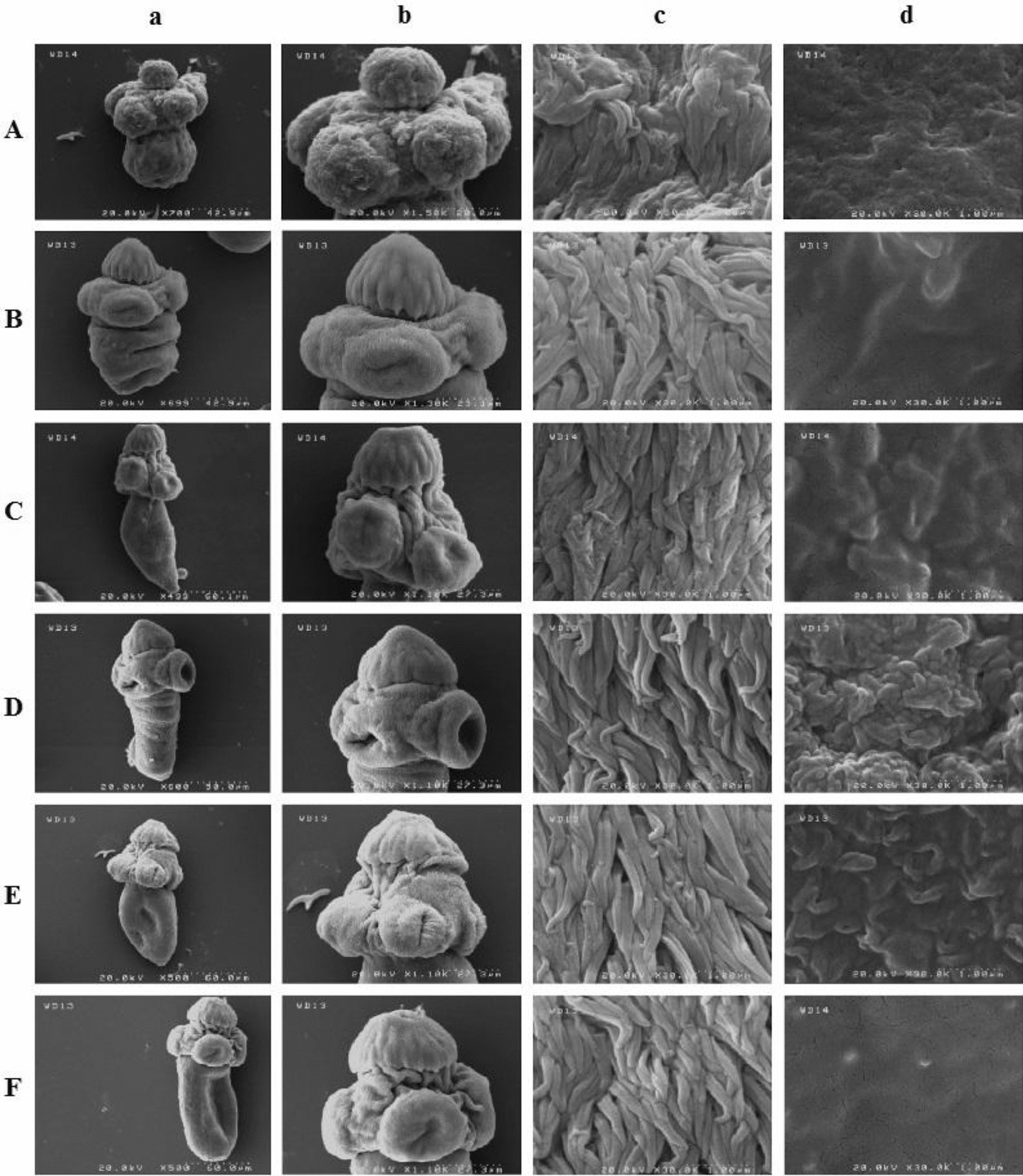
Scanning electron microscopy images of *E. granulosus *(*s.s.*) protoscoleces exposed for 24 h to 250 μM of the different drugs, including **A** tropisetron, **B** granisetron, **C** medium control, **D** CsA, **E** ABZSO and **F** DMSO control. **a** Complete protoscolex (magnification ×500), **b** sucker (magnification ×1100), **c** microtriches (magnification ×30,000) and **d** soma region (magnification × 30,000)

The PSC exposed to tropisetron exhibited a number of alterations, including rostellar disorganization, loss of hooks (Fig. 3Ab), destruction or shedding of microtriches (Fig. 3Ac) and contraction and marked tegumental change in the soma region (Fig. 3Ad). In those PSC exposed to granisetron, the tegument of the scolex and soma region remained unchanged (Fig. 3Bb), with regularity of the microtriches (Fig. 3Bc) and smoothness of the soma region (Fig. 3Bd). The PSC exposed to CsA showed marked alteration of the microtriches (Fig. 3Dc) and formation of tegumental vesicles in the soma region (Fig. 3Dd). In ABZSO-treated PSC, the microtriches remained stable (Fig. 3Ec); however, compared with those of the DMSO control group, minor alterations in the soma region were noted (Fig. 3Ed). In the PSC of the medium control (Fig. 3C) and the DMSO control (Fig. 3F), no significant alterations were visible in the tegument of the scolex or in the soma region was.

### Analysis of calcineurin A, calcineurin B, and calmodulin gene expression

**Fig. 4:**
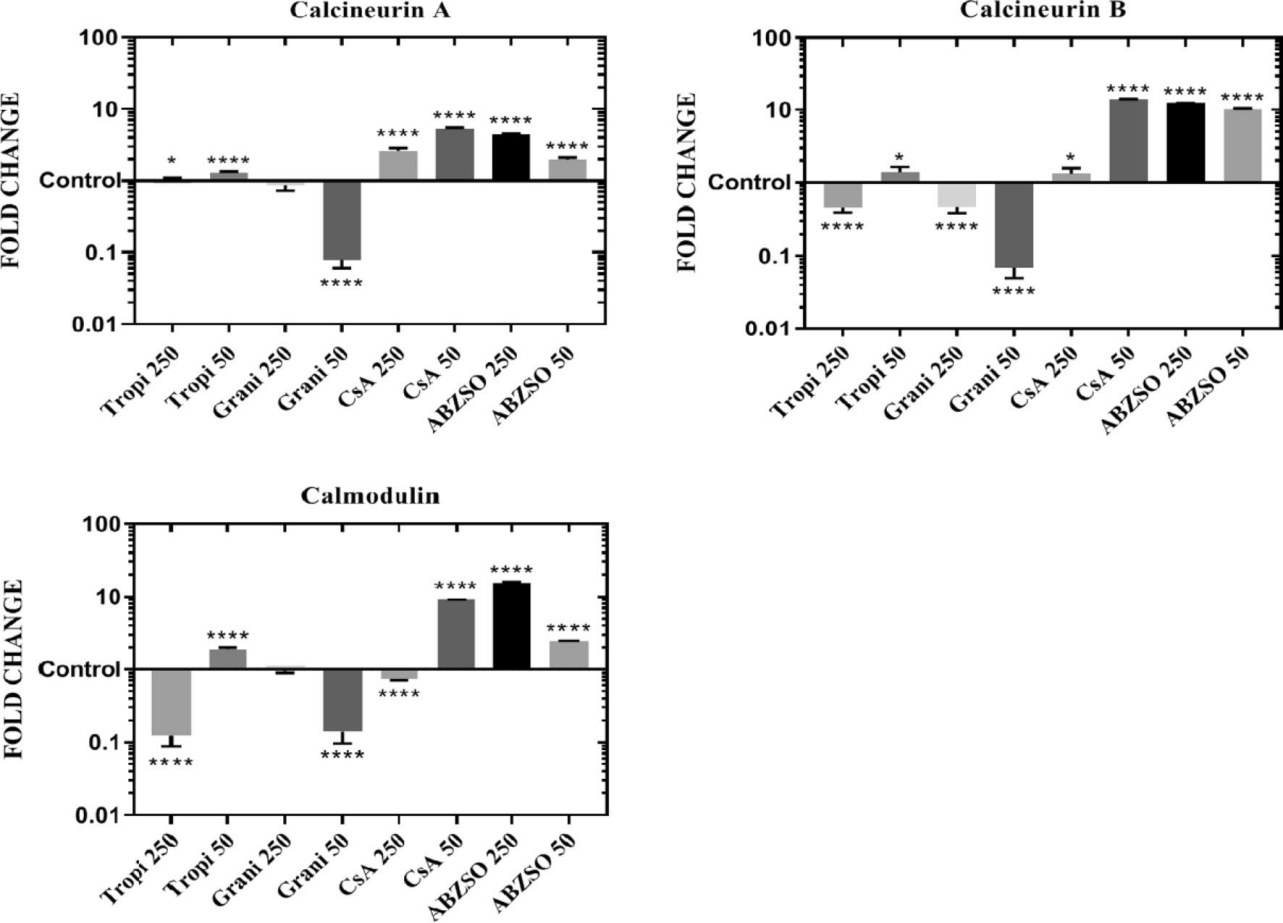
Gene expression analysis of *E. granulosus *(*s.s.*) PSC exposed to two concentrations (50 and 250 μM) of the different drugs in comparison with the control. The evaluated genes were calcineurin A, calcineurin B and calmodulin. The vertical axis of the charts represents the levels of mRNA expression (fold change: log10) in the exposed PSC and in the control group (fold change: 1). Fold change was calculated using REST 2009. The horizontal bars represent the mean ± standard deviation of three repetitions of triplicate experiments. The 2^−△△CT^ was the method of analysis. Asterisks indicate statistically significant differences from the control at *P < 0.05 and ****P < 0.0001, *Tropi* Tropisetron,* Grani* granisetron, *CsA* cyclosporine A, *ABZSO* Albendazole sulfoxide

In the PSC exposed to tropisetron, the expression levels of both the Eg-CaN-B and Eg-CaM genes were upregulated at 50 μM tropisetron and downregulated at 250 μM (Fig. 4). However, the expression of all three genes was downregulated at both of these concentrations in the PSC exposed to granisetron, in contrast to PSC exposed to ABZSO, which exhibited upregulation of all three genes at both concentrations. As for CsA, apart from the downregulation of Eg-CaM at 250 μM, only upregulations were noted. *P* values applicable to differences in gene expression changes are provided in Fig. 4.

### Discussion

In this study, we showed that both tropisetron and granisetron (5-HT_3_ antagonists) have dose-dependent protoscolicidal effects, but that this effectiveness is much stronger for tropisetron. The lack of an inhibitory effect of ondansetron, a selective 5-HT_3_ receptor antagonist, on *Echinococcus* motor activity induced by 5-HT or serotonin suggested that perhaps additional 5-HT receptors in the parasite are involved [31]. In the same way, it is assumed that the current results on the significant difference between the protoscolicidal effect of granisetron and tropisetron is most probably due to the dual molecular signaling pathway [9] of tropisetron and its function through a pathway other than 5-HT_3_. Therefore, further studies are necessary to determine the exact mechanism of the robust protoscolicidal effect of tropisetron.

With regard to CsA, its in vitro protoscolicidal effect has already been confirmed [21, 32]. The results of our study also indicated that it had a dose-dependent protoscolicidal effect at the same dose previously evaluated, although earlier studies reported a stronger protoscolicidal effect than observed in our study [32, 33]. This difference is assumed to be related to protoscolex activation prior to the drug exposure in the previous studies [32, 33], which was not performed in our study.

Albendazole is a drug whose protoscolicidal effects are well known, and it is currently used for CE chemotherapy [22]. Consistent with previous studies [34], our study also demonstrated the protoscolicidal effects of ABZSO, although it was less effective than tropisetron at all three administrated concentrations. It has been reported that praziquantel (PZQ) has a stronger in vitro protoscolicidal effect than ABZSO [35], but the* in vivo* use of PZQ was found to be less successful than albendazole [36], leading to the postulation that prior damage to the cyst wall by albendazole might facilitate the penetration and action of PZQ [37]. In the same way, the in vivo optimum duration and benefits of tropisetron as monotherapy and in combination with ABZSO and with PZQ in the treatment of CE should be determined in future studies.

One of the most conspicuous morphological changes observed in the drug-exposed PSC in our study was their evagination. The examination of morphological alterations in the parasites during exposure to the three different concentrations of the four study drugs over various periods of time indicated that the more rapid the evagination of PSC, as observed in those exposed to tropisetron, the faster their death. In contrast, continued invagination was associated with longer parasite viability. In one study involving in vitro exposure to CsA, invaginated PSC exhibited longer survival than evaginated ones that are highly motile and possibly take up and metabolize CsA at a faster rate than the relatively invaginated PSC [32]. Since one of the functions of the microtriches is nutrient absorption [38], drugs that rapidly trigger evagination may have a relatively stronger effect.

Another morphological change observed in the treated PSC was the presence of blebs at the tegument surface. We noted the formation of protoscolex blebs in various shapes and sizes, which developed simultaneously with other alterations. The development of blebs in the forms of small blebs (blister) or ballooning blebs in PSC exposed to PZQ has also been reported [39]. Bleb formation is assumed to reflect “stress responses” brought about in PSC by any harmful condition [34]. It has also been suggested that the presence of blebs might reflect a membrane repair mechanism in response to drug-induced damage or host antibody attacks [40]. Considering the variations in the size and shape of the blebs, it is possible that their development might rely on different mechanisms, but this hypothesis calls for further research.

SEM-based ultrastructural studies of PSC exposed for 24 h to 250 µM concentrations of the drugs revealed that the PSC incubated with granisetron failed to display visible changes in the tegument of the scolex and in the soma region. ABZSO induced slight alterations on the tegument of the soma region only, without visible changes in the microtriches. In the same way, PSC exposed to CsA revealed marked alterations of microtriches on the scolex region together with the formation of tegumental vesicles in the soma region. In contrast, tropisetron triggered pronounced alterations in the tegument of both the scolex and the soma region. In an earlier SEM-based ultrastructural study [34], exposure of PSC to ABZSO at 10 μg/ml resulted in pronounced bleb formation on the tegument of the soma region and the loss of hooks and shedding of microtriches in the scolex region; it should be emphasized, however, that such alterations were noted by the authors only after an incubation period of 24 days. Moreover, in another SEM study [41], the protoscoleces incubated* ex vivo* with 10 μg/ml of flubendazole for 6 days exhibited contraction and tegumental alterations of the soma region, while those incubated with nitazoxanide at 10 μg/ml for 3 days exhibited shedding of microtriches in the scolex region in addition to contraction of the soma region with marked alterations of the tegument. These results lead to the suggestion that the actions of the various chemotherapy agents are time- and dose-dependent; those agents that induce changes in the scolex, such as shedding of the microtriches, apparently have stronger protoscolicidal effects. The results of the present study illustrating the rapid and pronounced tegumental alteration of the PSC by tropisetron is an important new finding, which should stimulate further assessments of the protoscolicidal effectiveness of this compound.

The activity of calcineurin is affected by changes in intracellular Ca^2+^ [10]. This enzyme, in contrast to the many Ca^2+^-dependent kinases, is the only known protein phosphatase under the control of this cation and CaM [14]. A study on calcineurin expression in larval stages of *E. granulosus * (*s.l.*) also showed that Eg-CaN-B transcripts were downregulated in response to low intracellular Ca^2+^ levels; however, no change was found in the transcriptional level of Eg-CaN-A [14].

In the present study, gene expression was investigated for Eg-CaN-A, Eg-CaN-B and Eg-CaM after a 24-h exposure to granisetron, CsA, tropisetron and ABZSO, respectively, at concentrations of 50 and 250 μM. The results can be summarized as follows: (i) granisetron at both concentrations decreased the expression of Eg-CaN-B. As serotonin can increase intracellular calcium through 5-HT receptor activation [42], it is supposed that granisetron may act as a serotonin receptor antagonist rather than a 5-HT_3_ receptor, and that when intracellular calcium levels are not increased, a reduction in CaN-B activity and CaN-B gene downregulation is observed. In our study, CsA, in contrast to granisetron, increased Eg-CaN-B expression at both 50 and 250 μM. This drug increases intracellular calcium through inositol trisphosphate (InsP_3_)-dependent Ca^2+^ release, and calcineurin provides a negative feedback on InsP_3_/Ca^2+^signaling in blowfly salivary glands [43]. Therefore, we postulate that in the current study, CsA probably increased intracellular calcium by inhibiting this enzyme, leading to an increase in Eg-CaN-B gene expression. Tropisetron exhibited contrasting effects on Eg-CaN-B gene expression at both 50 and 250 μM. Unlike granisetron and CsA, tropisetron triggered decreased Eg-CaN-B expression in PSC exposed to 250 μM, but increased gene expression when exposed to 50 μM. However, the extent of gene expression decrease and increase was more limited than that of corresponding concentrations of granisetron and CsA, respectively. We assume that this dual effect of tropisetron might reflect its function either as a 5-HT antagonist, which does not increase intracellular calcium, or as an inhibitor of the calcineurin phosphatase enzyme, which increases intracellular calcium levels. However, further studies are required to enable more precise speculations on this interesting finding and the exact mechanism of tropisetron.

Exposure to 50 and 250 μM ABZSO resulted in an increased expression of the Eg-CaN-B gene. Since the calcineurin phosphatase enzyme is involved in tubulin polymerization [44] and albendazole inhibits beta-tubulin polymerization [45], it is likely that levels of phosphorylated tubulin dimers, as substrates of the CaN enzyme, are increased by the effect of ABZSO* via* inhibition of tubulin polymerization, which thereby results in CaN enzyme consumption and subsequent increased Eg-CaN-B gene expression.

The most important limitation of the present study was the lack of in vivo experiments to verify whether the drugs can pass through CE barriers. We did not establish an infection model for drug response monitoring. In line with the strategy of drug repurposing, the main strength of the study was the evaluation and documentation of the in vitro efficacy of tropisetron. This finding paves the way for evaluation of its application as a novel protoscolicidal agent during CE surgery and PAIR approaches informed by the results of future in vivo research.

### Conclusion

To summarize, among the four drugs we tested in vitro on PSC of *E. granulosus *(*s.s.*), tropisetron had the most potent effects, leading to evagination of PSC more rapidly than the other administered drugs. In line with the urgent need for introducing alternative chemotherapy options for CE, this important outcome should stimulate further in vitro studies on the mechanism of actions and in vivo evaluation of tropisetron effectiveness. Since this compound is currently considered to be a safe drug in clinics, its usefulness as a protoscolicidal agent in CE surgery and PAIR approaches should be evaluated. Moreover, further studies on the potential therapeutical effects of tropisetron on CE in experimentally infected laboratory animals as well as in naturally infected ruminants are recommended.

## Data Availability

The relevant information has been included in the manuscript. The datasets used and/or analyzed during the current study are available from the corresponding author on reasonable request.

## Abbreviations

ABZSO: Albendazole sulfoxide
BMZ: Benzimidazole
CaM: Calmodulin
CaN: Calcineurin
CE: Cystic echinococcosis
CsA: Cyclosporine A
DMSO: Dimethyl sulfoxide
GAPDH: Glyceraldehyde 3-phosphate dehydrogenase
5-HT_3_: 5-Hydroxytryptamine (serotonin) 3
PAIR: Puncture, aspiration, injection of the protoscolicidal agent, re-aspiration
PSC: Protoscoleces
SEM: Scanning electron microscopy
